# The Impact of Human Milk on Necrotizing Enterocolitis: A Systematic Review and Meta-Analysis

**DOI:** 10.3390/nu12051322

**Published:** 2020-05-06

**Authors:** Emma Altobelli, Paolo Matteo Angeletti, Alberto Verrotti, Reimondo Petrocelli

**Affiliations:** 1 Department of Life, Health and Environmental Sciences, University of L’Aquila, 67100 L’Aquila, Italy; paolomatteoangeletti@gmail.com; 2 Department of Pediatrics, University of L’Aquila, 67100 L’Aquila, Italy; alberto.verrottidipianella@univaq.it; 3 Public Health Unit, ASREM, 86100 Campobasso, Italy; reimondo.petrocelli@asrem.org

**Keywords:** human milk banks, NEC, meta-analysis, breast-feeding

## Abstract

Background. Premature infants receiving breastfeed have a lower incidence of NEC than those fed preterm formula. This study aimed: (1) to update a systematic review and meta-analyses to evaluate the relationship between feeding and necrotizing enterocolitis (NEC) in low weight premature infants; (2) to conduct meta-regression analyses by subgroups; (3) to describe geographical distribution of milk banks in the world. Methods. Papers included in the meta-analysis were updated as of June 2019. Relative risks were used as a measure of effect size. Random effect models were used to account for different sources of variation among studies. For milk banks, the data reviewed by the literature were integrated with the information collected from countries’ institutional sites and milk bank networks. Results. Thirty-two papers were included in meta-analysis: six randomized controlled trials (RCTs) and 26 observational studies (OS). The census has found 572 milk banks around in the world. Brazil has the most active milk banks. RCTs meta-analysis indicates a risk reduction of NEC using human milk respect to formula: Relative risk (RR) = 0.62 (0.42–0.93). Seven OS compared quantities lower than human milk or higher than the 50th quantile showing a risk reduction of NEC:RR = 0.51 (0.31–0.85); 3 OS that evaluated human milk versus mixed feeding showing that human milk has a protective role on the development of NEC:RR = 0.74 (0.63–0.91). Results of subgroups analysis show that the risk reduction is statistically significant only for studies in which premature infants are given both their own and donated breastmilk. Conclusions. The possibility of preserving human milk and promoting donations guarantees an improvement in the health of newborns.

## 1. Introduction 

Necrotizing enterocolitis (NEC) is the most devastating intestinal disease in neonates with very-low-birth-weight (VLBW). Incidence varies in different studies. Yee et al. [1] and Fitzgibbons et al [2] showed, in large cohort studies of VLBW infants, respectively, NEC incidence from 1.3% to 12.9% and from 3% to 12%; while more recently observational studies showed an incidence from 20.7% [3] to 16.7% [4,5]. 

It is universally accepted that human milk is the optimum source of nutrition for the first six months of life. The health benefits of human milk are known, not only for premature infants [6], but also for prevention of other infant diseases [7]. 

Premature infants receiving human milk have a lower incidence of NEC than those fed preterm formula [8]. In fact, some studies suggest that mother’s milk is protective against sepsis, because it contains bioactive substances that have bactericidal and immune-modulating activities [9].

Shoji et al. support the hypothesis that breastmilk has antioxidant proprieties with a protective effect from oxidative stress [10]. In this contest, it is important to underline that not all mothers produce sufficient milk for their neonate, and donor human milk (DM) has been considered as an alternative to mother’s milk (MM) [11]. Human milk inhibits the growth of *Escherichia coli, Staphylococcus aureus* and *Candida* sp. [12]. DM is generally pasteurized to prevent the potential risk for the transmission of pathogens from donor mothers to preterm infants. Its safety must be considered accurately because pasteurization reduces the concentration of immunological proteins in human milk [13,14]. Human milk banking is an absolute necessity, especially for premature infants. Unfortunately, human milk banks are not present in each country of the world. Infants can suffer the consequences if there are no banks. Generally, the collection and processing of human milk is established by guidelines. Globally, there is a growing interest to increase milk banks by also raising awareness campaigns to donate milk.

The aims of our study are: (i) to update a systematic review and meta-analyses of observational and RCT studies that evaluated the possible relationship between feeding (maternal, preterm formula, mixed maternal-formula) and development of necrotizing enterocolitis (NEC) in premature infants low weight; (ii) to conduct meta-regression analyses evaluating continuous and geographical variables; (iii) to describe the geographical distribution of milk banks in the world.

## 2. Materials and Methods 

The papers included in the meta-analysis were sought in the MEDLINE, EMBASE, Scopus, Clinicaltrials.gov, Web of Science, and Cochrane Library databases as of June 2019. The search terms used were milk, human OR breast feeding OR milk banks OR breast milk expression OR breastfeed* OR breastfed OR breast OR HM OR fed OR feed* OR enteral nutrition AND enteral nutrition AND infant, premature OR infants, extremely premature OR infant, low birth weight OR infant, very low birth weight OR intensive care units, neonatal OR intensive care, neonatal OR premature birth AND low birthweight OR low birth weight OR VLBW OR ELBW OR Prematur* OR Preterm OR pre-term OR infant* OR newborn* OR new-born* OR baby* OR babies OR neonatal intensive care OR NICU AND enterocolitis, necrotizing. Filters: Filters: 15 years, Humans, Child: birth-18 years.

**Figure 1:**
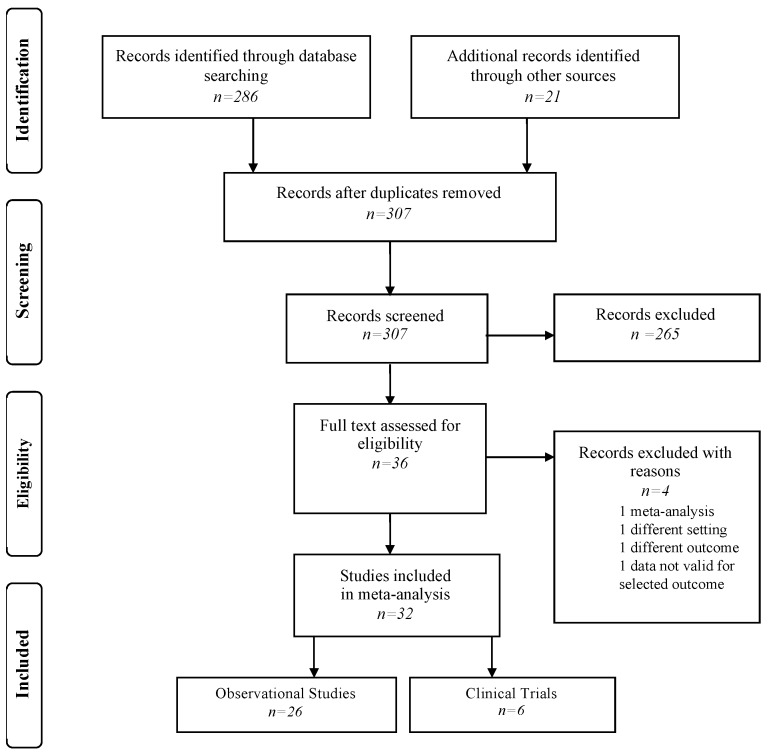
Flow-chart of search strategy.

Papers were selected using the Preferred Reporting Items for Systematic Reviews and Meta-Analyses (PRISMA) flowchart (Figure 1) and the PRISMA checklist (Table S1) [15]. A manual search of possible references of interest was also performed. Only studies published in English over the previous 15 years were considered. The papers were selected by two independent reviewers (P.M.A. and A.V.); a methodologist (E.A.) resolved any disagreements. Bias was assessed using the Cochrane Collaboration tool for assessing risk of bias and the Newcastle–Ottawa scale for cohort studies (Tables S2 and S3) [16,17]. 

For the research of milk banks, place where HM is collected and/or treated and/or distributed, the data reviewed by the literature were integrated with the information collected from the institutional sites of the individual countries and from European and North American milk bank networks. Other information sources have been obtained from the literature using the following words: “human milk banks” AND “state name”. Moreover, we used the information obtained from the institutional sites for each country [18,19]. Data on premature births were derived from WHO datasets [20]. The ratio between milk banks and premature births per 100,000 was performed.

### Statistical Analysis

We consider NEC if patients had a Bell’s score ≥2. Relative risks (RRs), with 95% CI and p-value, were used as a measure of effect size. Random effect models were used to account for different sources of variation among studies. Heterogeneity was assessed using Q statistics and I^2^. The stability of study findings was checked with moderator analysis. Publication bias was analyzed and represented by a funnel plot, and funnel plot symmetry was assessed with Egger’s test [21,22]. Publication bias was checked using the trim and fill procedure [22,23]; PROMETA 3 software (IDo Statistics-Internovi, Cesena, Italy) was used. Finally, meta-regression analyses, using a random effects model, were utilized for the following variables: publication year of article, gender, birth weight and gestational age and geographical area; for continuous variables, regression models were used; for categorical variables, an ANOVA-Q test was used. Meta-regressions were performed when the number of studies containing the variables to be analyzed was ≥3. Meta-analyses and meta-regressions were conducted according to the study design: randomized and observational studies. In the context of observational studies, a distinction was made based on the type of comparison HM vs mixed feeding, HM vs only preterm formula and mixed feeding vs only preterm formula. The articles presenting data in quantiles of consumed milk were analyzed by dichotomically dividing the patients into two groups on the basis of the presence of human milk in their diet: inferior or superior to the 50° quantile. A subgroup analysis was also conducted, excluding studies that, in the preterm formula group, had an incidence of NEC above 15%. 

## 3. Results

### 3.1. Literature Search Results 

Research has highlighted the presence of 307 records. In the screening phase, 271 references were excluded; therefore, 36 full texts were considered. Of these, 4 were not considered for different reasons: one paper was a meta-analysis [23], one was an RCT that reviewed early progressive feeding [24], one had another outcome regarding neurological follow-up of premature children respect to the nourishment adopted [25], one paper analyzed only children that took maternal milk or formula or donated milk the same or more than 50° quantile not allowing consistent comparisons with the other primary studies selected [26]. Finally, 32 papers were included in quantitative analysis: six clinical trials [6,27,28,29,30,31,32] and 26 observational studies [3,4,5,33,34,35,36,37,38,39,40,41,42,43,44,45,46,47,48,49,50,51,52,53,54]. 

The characteristics of the studies, compared to the type of human milk adopted, are reported in the Tables S4, S5. The characteristics concerning the population of the single studies are reported in the Table S6.

### 3.2. Meta-Analysis and Meta-Regression Results

Selected trials [6,27,28,29,30,31] investigated the occurrence of NEC in breastfed premature infants compared to those fed preterm formula. Their total sample size is 1626 newborns. The meta-analysis indicates that there is a clear indication of risk reduction in the use of human milk respect to formula, RR = 0.62 (0.42–0.93), and this result occurs in presence of statistical heterogeneity among primary studies analyzed (I^2^ = 47.03, *p* = 0.009) (Table 1, Figure S1). 

The meta-regression analyses by year of publication, male gender, birth weight, gestational age and ethnicity do not show statistically significant results; in fact, it is important to underline that these variables do not influence NEC incidence (Table 2). Trim and fill analyses do not show differences among observed and estimated values and any studies were trimmed. 

Eighteen observational studies [3,4,5,32,33,34,35,36,37,38,39,40,41,42,43,44,45,46,47] investigated the comparison between human milk vs formula for a total of 6,405 newborns. The overall result indicates that there is a reduction in the risk of NEC, RR = 0.45 (0.32–0.62, *p* < 0.001), with statistically significant heterogeneity among primary studies analyzed (I^2^ = 55.25, *p* = 0.002) (Table 1, Figure S1). 

The meta-regression analyses by year of publication, gender, birth weight, gestational age and ethnicity does not show statistically significant results (Table 2). The analysis by geographical area shows statistically significant differences, with risk reduction in Europe and USA, but not in Japan, probably because there is only one study with a small sample (Table 3). 

Seven observational studies [49,50,51,52,53,54,55] compared high consumption of human milk against low consumption for a total of 2,453 newborns. The results show a risk reduction of NEC, RR = 0.51 (0.31–0.85, *p* = 0.01), without statistically significant heterogeneity (I^2^ = 9.21, *p* = 0.359) (Table 1, Figure S1).

The meta-regression analyses show that there are no significant results regarding the year of publication, birth weight and ethnicity, but they show a statistically significant result with regard to male gender (Table 2). 

Three studies [35,36,37] that evaluated breastfeeding versus mixed feeding (Table 1) show that human milk could have a protective role on the development of NEC, RR = 0.74 (0.63–0.91, *p* = 0.003), both without statistically significant heterogeneity (I^2^ = 0.00, *p* = 0.407) and in the absence of publication bias (Table 1, Figure S1). 

The meta-regression analyses for publication year and birth weight do not show statistically significant results (Table 2).

Finally, four studies concerned the comparison between mixed feeding and preterm formula (Table 1) [35,36,37,38]. In these it is revealed that mixed feeding is a risk factor for the development of NEC, RR = 1.37 (1.13–1.65, *p* = 0.001), without statistically significant heterogeneity (I^2^ = 0.00, *p* = 0.774) and publication bias (Table 1, Figure S1).

The meta-regression analysis for year of publication shows a statistically significant result (Table 2), but not for birth weight. 

Finally, trim and fill analysis do not show differences among observed and estimated values and any study were trimmed.

### 3.3. Human Milk Bank for Premature Birth

**Figure 2:**
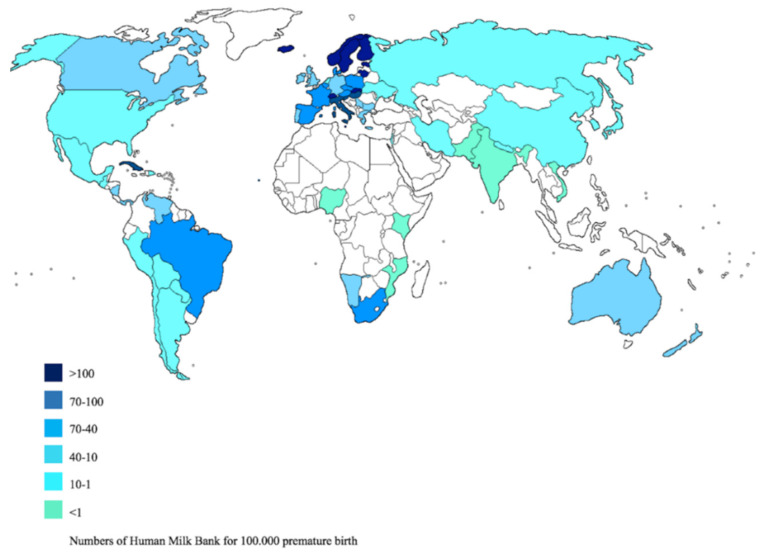
Distribution of Human Milk Bank in the world.

The census of milk banks has found in the world 572 milk banks, not evenly distributed and were reported on Table S7. Brazil holds the record for the number of active milk banks (214), followed by South Africa with 44, Italy with 37 banks and in Europe 238. Norway, Sweden, Finland, Estonia, Switzerland, Slovakia and Cuba have the largest number of milk banks per premature baby (Figure 2).

## 4. Discussion

Some fundamental aspects emerge from our work: the first is that human milk, breastfeed or donor, has a protective effect in the development of NEC in premature babies: in fact, it can be seen that the use of human milk is a protective factor in the development of NEC in premature babies; the second underlines that there is no homogeneous distribution of the places of the milk banks where HM is collected and/or pasteurized and/or distributed. The latter represents a limitation of the study: particularly the pasteurization, with thermal damage, reduces microbial contamination and immunological components [9]. A second limitation is that, considering the primary studies, it was not possible to extrapolate the policies relating to the implementation of the milk banks; in addition, breast milk and donated milk were considered as a single entity of comparison with breastfeeding with formula, although statistically there is no evidence for publication bias.

A third limitation consists in the fact that the countries considered are very heterogeneous, both from the socio-cultural point of view and from the point of view of organizing health systems. In addition, it should be emphasized that, even within the same state, there are differences between the north and south. Heterogeneity between countries and between north and south of the same country can present socio-cultural differences. There may be religious differences between one state and another or between one continent and another. Another fundamental difference is represented by the income. 

Finally, some works [3,4,5,6,31] show a higher incidences of NEC compared to the others. This could partially justify the clinical heterogeneity of the studies, although the analysis of the risk of bias does not highlight substantial critical issues.

The United Nations Objective 3.4 for “Sustainable development is linked to the reduction of premature mortality” [55]. The guidelines of the WHO and the main American and European scientific associations indicate that, in the absence of breast milk, donated milk is the second food of choice [56]. Where breastfeeding is not possible, donated milk stored in milk banks can be used [57,58,59,60]. Our work highlighted that there is an inhomogeneous distribution of milk banks (Figure 2).

It has been estimated that 52.9% of premature births occur in Asia, 25% in sub-Saharan Africa, 7.7% in Latin America, 5.7% in Europe, 4.1 % in North Africa, 3.1 in North America and 0.5% in Oceania. This data obviously reflects differences in terms of methodological qualities between countries with high and low incomes [61]. A European study shows that the costs of hospitalization of a premature are 10,000 euros more than a not premature one [62]. Regarding milk banks, in Asia and Africa there is a lower presence (more concentrated only in some countries) both in percentage terms and in relation to the number of premature per 100,000 births. This data shows that there are inequalities in access to donated breast milk.

A liter of donated milk has been estimated 82.88 euro in Germany [63], while in the US the costs is 150 dollars [64,65]. 

It is important to underline that the administration of donated breast milk, as we have seen, associated with the continuous improvement of neonatal techniques, could significantly reduce the costs of hospitalization and assistance in the short term.

A study conducted in the US shows that treatment with breast feed fortifier derived from human donated milk in the prevention of NEC is cost effective with respect to breast feed fortifier derived from bovine milk [66]. This effect could be very important in the long run. It is known that breast milk improves neuro-cognitive development, potentially also reducing the costs of social and scholastic assistance linked to any deficit, even if there are no studies on the subject to date. Therefore, the establishment of a milk bank does not appear to be anti-economical, but rather as a precise choice in maternal and child health policies aimed at reducing social inequalities. Online sales of breast milk are also described, but being out of the milk bank circuit, do not have the same quality standard [67]. Within the advanced countries there are differences in the distribution of milk banks: for example, in Italy there is a difference between the north and south, being more widespread in the north of the country with a higher income to the detriment of the southern areas with a lower income [68]. At the same time, the scarcity of the resource imposes choices with respect to whom should administer this good.

Our results show that feeding with breast milk reduces the risk of NEC. In particular, it is noted that in studies that compared the low versus high consumption of their own breast milk, there is a significant reduction in the risk of NEC. This information is also confirmed in observational studies and in clinical trials in which human milk (donated or own) is compared with formulas: in both cases there is a risk reduction. In addition, subgroup analysis is particularly interesting. It shows that the risk reduction is statistically significant only for studies in which premature are given both their own and donated breastmilk. It should be emphasized that heterogeneity is statistically significant in the absence of publication bias. The nature of these results could be explained by the difference between maternal milk and donated maternal milk, which undergoes pasteurization processes to eliminate possible pathogens. In fact, in a recent study a difference of macronutrients and proteins is highlighted: the donated milk would have less grams of protein per deciliter (1.42 vs. 1.52) and less fat content per deciliter (3.41 vs. 3.79), therefore a lower energy content (63.80 vs. 67.29) [58]. However, the pasteurization of breast milk appears to be a hygiene practice of fundamental importance in order to eliminate the bacterial and viral load, despite sacrificing some beneficial components in breast milk [58,59]. Alternative methods to pasteurization are being studied to preserve all the qualities of breast milk [58]. 

The variables investigated with meta-regression analysis do not provide explanations on the factors that may affect the risk of NEC, with the exception of publication year and male gender. In the first case, this result could be justified by an overall improvement in the health care of premature neonates, as highlighted also in the most recently published papers [28]. In the second, the result could be influenced by selection methods of the patients, not being known in the literature a greater predisposition of male gender on the incidence of prematurity. 

Furthermore, it is important to remember that the use of the random effects model, also for meta-regressions, allows to ascertain the possible epidemiological link between the observed effect and the variables investigated; this aspect is strengthened in the absence of statistically significant publication bias. 

## 5. Conclusions 

In conclusion, our study shows a clear benefit of breastfeeding or, in its absence, with donated milk and highlights a heterogeneity in the distribution of milk banks between countries and within the same country. Particularly in Africa, the Middle East and Asia, where Muslim populations are dominant. In addition, our results underline the relationship between feeding and development of NEC [69]. 

It therefore highlights the potential benefit of accessing a resource, breast milk, appropriately stored at the milk banks. In addition, this breastfeeding should also be encouraged in order to reduce the impact of other pathologies related to lactation [23]. Failure to access socio-health infrastructures such as milk banks could create inequalities for the prevention of a high mortality disease. Further investigations, which go beyond the objectives of this meta-analysis, should be addressed on the link between NEC and pasteurized or unpasteurized human milk.

Finally, almost all primary studies have been conducted in Europe and the United States. The possibility of preserving breast milk and promoting donations (implicitly also supporting control over maternal health conditions, promoting virtuous behavior) guarantees an improvement in the health of newborns. Of particular interest would be to evaluate the incidence of NEC in Arab countries, where breastfeeding is abandoned early and the donation of breast milk is not particularly successful for religious reasons.

International cooperation and the authorities of the single countries should provide some targeted interventions for the realization of milk banks that, in the last analysis, represent a fortress of health and social justice. In particular, the use of donor milk is widely endorsed.

## Figures and Tables

**Table 1:** Meta-analysis results.

	Pooled Analysis		Heterogeneity		Publication Bias			
	RR (95% CI)	*p*-Value	I^2^	p-Value	Egger’s Test		Begg’s and Mazdumdar’s Tests	
					T	*p*-Value	Z	*p*-Value
RCT								
Human milk (breastfeeding and donor) vs preterm formula *k* = 6 [6,27,28,29,30,31]	0.62 (0.42–0.93)	0.02	47.03	0.009	−1.82	0.144	−2.44	0.015
Human milk (breastfeeding and donor) vs preterm formula *k =* 4 * [27,28,29,30]	0.57 (0.32–1.01)	0.054	64.01	0.040	−1.64	0.243	−2.04	0.174
Observational studies								
>50° quantile of human milk of total enteral feeding *k =* 7 [48,49,50,51,52,53,54]	0.51 (0.31–0.85)	0.001	9.21	0.359	−2.02	0.078	−02.27	0.788
Human milk (breastfeeding and donor) vs preterm formula *k =* 18 [3,4,5,32,33,34,35,36,37,38,39,40,41,42,43,44,45,46,47]	0.45 (0.32–0.62)	<0.001	55.25	0.002	−0.35	0.731	0.11	0.910
Human milk (breastfeeding and donor) vs preterm formula *k =* 15 [32,33,34,35,36,37,38,39,40,41,42,43,44,45,46,47]	0.45 (0.30–0.69)	<0.001	56.61	0.004	−0.97	0.35	0.35	0.729
Human milk (breastfeeding and donor) vs mixed feeding *k =* 3 [35,36,37]	0.74 (0.63–0.91)	0.003	0.00	0.407	0.11	0.925	−0.68	0.497
Mixed feeding vs preterm formula *k* = 4 [37,38].	1.37 (1.13–1.65)	0.001	0.00	0.774	0.23	0.871	0.00	1.00

Legend: RCT: randomized controlled trial; RR: relative risk; CI: confidence interval; k: numbers of primary studies *Excluding paper reporting NEC (necrotizing enterocolitis) incidence >15% of in preterm formula groups.

**Table 2:** Meta-regressions: results for continuous variables.

	No. of Primary Studies	Total Sample Size	Intercept (y)	Slope (x)	*p*-Value
RCT					
Human milk vs preterm formula					
Publication year	6	1626	−107.64	0.05	0.472
Male (%)	4	775	−2.96	0.04	0.383
Birth weight	4	1084	−5.21	0.00	0.068
Gestational age	5	1253	−5.21	0.26	0.393
Caucasians (%)	3	1030	−0.20	−0.02	1.186
Observational Studies					
High vs low dose					
Publication year	7	2453	21.34	−0.02	0.883
Male (%)	5	1950	7.84	−0.18	0.039
Birth weight	3	835	2.88	0.0	0.189
Caucasians (%)	4	1982	2.22	−0.05	0.064
Human milk vs preterm formula					
Publication year	18	6405	145.52	−0.07	0.077
Male (%)	15	4730	0.16	−0.02	0.682
Birth weight	14	5424	0.63	0.00	0.234
Gestational age	11	2875	1.56	−0.08	0.680
Caucasians (%)	5	3558	−0.18	−0.02	0.096
Human milk vs mixed feeding					
Publication year	3	2071	190.88	−0.09	0.624
Birth weight	3	2071	−0.83	0.00	0.659
Mixed feeding vs preterm formula					
Publication year	4	2089	−110.88	−0.05	0.009
Birth weight	3	1708	−0.75	0.00	0.491

**Table 3:** Observational studies regarding human milk vs preterm formula: meta-regression results of geographical areas.

	No. of Primary Studies	Total Sample Size	RR (95% CI)	*p*-Value	I^2^	Q	*p*-Value	Overall ANOVA Q-Test
Europe	9	3398	0.53 (0.35–0.79)	0.02	35.82	12.31	0.138	0.42, *p* = 0.811
Japan	1	18	0.81 (0.01–45.22)	0.92	-	-	-	
USA	8	3876	0.43 (0.26–0.71)	0.71	72.19	25.17	0.001	

## Supplementary Materials

The following are available online at https://www.mdpi.com/2072-6643/12/5/1322/s1, Table S1: Prisma Checklist, Table S2: Trial bias assessment according to Cochrane Collaboration, Table S3 Observational Studies bias assessment according to New Castle-Ottawa scale, Table S4: Nutritional Pattern of Interventional Studies, Table S5: Nutritional Pattern of Observational Studies, Table S6: Characteristics of included studies, Table S7: Human Milk Banks in the world. Figure S1: Meta-analysis: results from RCT and observational studies. Forest plot for selected outcomes.
