# Supplementary material for: The Impact of Human Milk on Necrotizing Enterocolitis: A Systematic Review and Meta-Analysis
Source: Nutrients. 2020 May 6;12(5):1322. doi: 10.3390/nu12051322 (PMC7284425; doi:10.3390/nu12051322)
Supplement: Supplementary file 1 [file nutrients-12-01322-s001.pdf]

Supplementary Materials

Table S1. PRISMA Checklist.

Section/Topic	#	Checklist item	Reported on page #
		**TITLE**	
Title	1	Identify the report as a systematic review, meta-analysis, or both.	1
		**ABSTRACT**	
Structured summary	2	Provide a structured summary including, as applicable: background; objectives; data sources; study eligibility criteria, participants, and interventions; study appraisal and synthesis methods; results; limitations; conclusions and implications of key findings; systematic review registration number.	1-2
		**INTRODUCTION**	
Rationale	3	Describe the rationale for the review in the context of what is already known.	1-2
Objectives	4	Provide an explicit statement of questions being addressed with reference to participants, interventions, comparisons, outcomes, and study design (PICOS).	1-2
		**METHODS**	
Protocol and registration	5	Indicate if a review protocol exists, if and where it can be accessed (e.g., Web address), and, if available, provide registration information including registration number.	Not applicable
Eligibility criteria	6	Specify study characteristics (e.g., PICOS, length of follow-up) and report characteristics (e.g., years considered, language, publication status) used as criteria for eligibility, giving rationale.	2
Information sources	7	Describe all information sources (e.g., databases with dates of coverage, contact with study authors to identify additional studies) in the search and date last searched.	2
Search	8	Present full electronic search strategy for at least one database, including any limits used, such that it could be repeated.	2
Study selection	9	State the process for selecting studies (i.e., screening, eligibility, included in systematic review, and, if applicable, included in the meta-analysis).	2
Data collection process	10	Describe method of data extraction from reports (e.g., piloted forms, independently, in duplicate) and any processes for obtaining and confirming data from investigators.	2
Data items	11	List and define all variables for which data were sought (e.g., PICOS, funding sources) and any assumptions and simplifications made.	2
Risk of bias in individual studies	12	Describe methods used for assessing risk of bias of individual studies (including specification of whether this was done at the study or outcome level), and how this information is to be used in any data synthesis.	2
Summary measures	13	State the principal summary measures (e.g., risk ratio, difference in means).	2
Synthesis of results	14	Describe the methods of handling data and combining results of studies, if done, including measures of consistency (e.g., I^2) for each meta-analysis.	2
Risk of bias across studies	15	Specify any assessment of risk of bias that may affect the cumulative evidence (e.g., publication bias, selective reporting within studies).	2
Additional analyses	16	Describe methods of additional analyses (e.g., sensitivity or subgroup analyses, meta-regression), if done, indicating which were pre-specified.	2
**RESULTS**			
Study selection	17	Give numbers of studies screened, assessed for eligibility, and included in the review, with reasons for exclusions at each stage, ideally with a flow diagram.	3-10
Study characteristics	18	For each study, present characteristics for which data were extracted (e.g., study size, PICOS, follow-up period) and provide the citations.	3-10
Risk of bias within studies	19	Present data on risk of bias of each study and, if available, any outcome level assessment (see item 12).	3-10
Results of individual studies	20	For all outcomes considered (benefits or harms), present, for each study: (a) simple summary data for each intervention group (b) effect estimates and confidence intervals, ideally with a forest plot.	3-10
Synthesis of results	21	Present results of each meta-analysis done, including confidence intervals and measures of consistency.	3-10
Risk of bias across studies	22	Present results of any assessment of risk of bias across studies (see Item 15).	3-10

Additional analysis	23	Give results of additional analyses, if done (e.g., sensitivity or subgroup analyses, meta-regression [see Item 16]).	3-10
**DISCUSSION**			
Summary of evidence	24	Summarize the main findings including the strength of evidence for each main outcome; consider their relevance to key groups (e.g., healthcare providers, users, and policy makers).	10-12
Limitations	25	Discuss limitations at study and outcome level (e.g., risk of bias), and at review-level (e.g., incomplete retrieval of identified research, reporting bias).	10-12
Conclusions	26	Provide a general interpretation of the results in the context of other evidence, and implications for future research.	10-12
**FUNDING**			
Funding	27	Describe sources of funding for the systematic review and other support (e.g., supply of data); role of funders for the systematic review.	Not applicable

Table S2. Trial bias assessment according to Cochrane Collaboration.

Study	Random sequence generation (selection bias)	Allocation concealment (selection bias)	Blinding of participants and personnel (performance bias)	Blinding of outcome assessment (detection bias) (patient-reported outcomes)	Incomplete outcome data addressed (attrition bias) (Short-term outcomes (2-6 weeks))	Incomplete outcome data addressed (attrition bias) (Longer-term outcomes (>6 weeks))	Selective reporting (reporting bias)
**Cristofalo 2013**	**Low**	**Low**	**Low**	**Unclear**	**Low**	**Low**	**Low**
**O'Connor 2016**	**Low**	**Low**	**Not Applicable**	**Unclear**	**Low**	**Low**	**Low**
**Sullivan 2010**	**Low**	**Low**	**Not Applicable**	**Unclear**	**Low**	**Low**	**Low**
**Schanler 2005**	**Low**	**Low**	**Unclear**	**Unclear**	**Low**	**Low**	**Low**
**Manzoni 2013**	**Low**	**Low**	**Not Applicable**	**Unclear**	**Low**	**Low**	**Low**
**Corpeleijn 2016**	**Low**	**Low**	**Low**	**Unclear**	**Low**	**Low**	**Low**

Table S3. Observational Studies bias assessment according to New Castle-Ottawa scale.

**Study ID**	**Selection**				**Comparability**	**Outcome**		
	**Representativeness of exposed cohort**	**Selection of the non-exposed cohort**	**Ascertainment of exposure**	**Demonstration that outcome of interest was not present at the start of the study**	Comparability of the cohort on the basis of the design of analysis	Assessment of outcome	Was follow-up long enough for outcomes to occur	Adequacy of follow up cohorts
**Bishop 2010**	***A**	***A**	***A**	**B**	**A***	***A**	**A***	**B**
**Zamkir 2018**	***A**	***A**	***A**	**B**	**A***	***A**	**A***	**B**
**Spiegler 2016**	***A**	***A**	***A**	**B**	**A***	***A**	**A***	**B**
**Corpeleijin 2012**	***A**	***A**	***A**	**B**	**A***	***A**	**A***	**B**
**Berkhout 2018**	***A**	***A**	***A**	**B**	**A***	***A**	**A***	**B**
**Chowing 2016**	***A**	***A**	***A**	**B**	**A***	***A**	**A***	**B**
**Manzoni 2013**	***A**	***A**	***A**	**B**	**A***	***A**	**A***	**B**

**Colaizy 2012**	***A**	***A**	***A**	**B**	**A***	***A**	**A***	**B**
**Kreissl 2017**	***A**	***A**	***A**	**B**	**A***	***A**	**A***	**B**
**Jacobi Pollistok 2016**	***A**	***A**	***A**	**B**	**A***	***A**	**A***	**B**
**Paker 2012**	***A**	***A**	***A**	**B**	**A***	***A**	**A***	**B**
**Vohr 2007**	***A**	***A**	***A**	**B**	**A***	***A**	**A***	**B**
**Huston 2018**	***A**	***A**	***A**	**B**	**A***	***A**	**A***	**B**
**Giuliani 2012**	***A**	***A**	***A**	**B**	**A***	***A**	**A***	**B**
**Ginovart 2016**	***A**	***A**	***A**	**B**	**A***	***A**	**A***	**B**
**Tanaka 2009**	***A**	***A**	***A**	**B**	**A***	***A**	**A***	**B**
**Furman 2003**	***A**	***A**	***A**	**B**	**A***	***A**	**A***	**B**
**Madore2 017**	***A**	***A**	***A**	**B**	**A***	***A**	**A***	**B**
**Herrman 2014**	***A**	***A**	***A**	**B**	**A***	***A**	**A***	**B**
**Sisk 2007**	***A**	***A**	***A**	**B**	**A***	***A**	**A***	**B**
**Sisk 2016**	***A**	***A**	***A**	**B**	**A***	***A**	**A***	**B**
**Manea 2018**	***A**	***A**	***A**	**B**	**A***	***A**	**A***	**B**
**Chowing 2016**	***A**	***A**	***A**	**B**	**A***	***A**	**A***	**B**
**Maayan-Metzger 2012**	***A**	***A**	***A**	**B**	**A***	***A**	**A***	**B**
**Assad 2016**	***A**	***A**	***A**	**B**	**A***	***A**	**A***	**B**
**Giuliani 2012**	***A**	***A**	***A**	**B**	**A***	***A**	**A***	**B**

Table S4. Nutritional Pattern of Interventional Studies.

Author, year	Country	Study Polulation	Intervention	Control
**Cristofalo 2013**	USA and Austria	VLBW	Donor milk fortifier (20 kcal/oz Prolacta Bioscience, Monrovia)	Preterm Formula (24 kcal/oz)
**O'Connor 2016**	Canada	VLBW	Donor milk fortifier (Mother's milk bank of Ohio and NorthenStar Mother's milk bank with fortifier (Similiac Humal Milk Fortifier or Enfamil Human Milk fortifier)	Preterm Formula (Similac Special Care (Abbot) Enfamil Premature (Mead Johnson Nutritional) 20-20 Kcal/oz 3 gr protein/100
**Sullivan 2010**	USA and Austria	VLBW	Donor milk fortifier (Prolact+H2MF, Prolacta Bioscience)	Preterm Formula
**Schanler 2005**	USA	VLBW	Mother Milk or Donor milk (Mother milk bank, Presbyterian/-St Luke Medical center, Denver and Lactation Center WakeMed, Raleigh) and Mother milk fortifier (Enfamil Human Milk Fortifier Mead Johnson Nutritional)	Preterm Formula Enfamil Preamture Formula (100 kj/oz)
**Manzoni 2013**	Italy	VLBW	Human milk (mother and donor)	Preterm formula
**Corpeleijn 2016**	The Netherlands	VLBW	Donor Milk (Dutch Human Milk Bank)	Preterm formula (Nenatal Start Nutricia Advanced Medical Nutrition) or Hero Premature (Hero)

Table S5. Nutritional Pattern of Observational Studies.

Author, year	Country	Study Population	Group 1	Group 2	Group 3
**Bishop 2010**	USA	VLBW	Donor milk	Preterm formula	-
**Zamkir 2018**	Germany	VLBW	Human milk	Preterm formula	Mixed feeding
**Spiegler 2016**	Germany	VLBW	Human milk (mother and donor)	Preterm formula	Mixed feeding
**Corpeleijin 2012**	Netherlands	VLBW	Mother milk	Preterm formula	
**Berkhout 2018**	Netherlands and Belgium	Preterm	Human milk (mother and donor)	Preterm formula	Mixed feeding
**Chowing 2016**	USA	VLBW	< 50% Human Milk	>50% Human Milk	-
**Manzoni 2013**	Italy	VLBW	Human milk (mother and donor)	Preterm formula	-
**Colaizy 2012**	USA	VLBW	Donor Milk (different percentiles)	Preterm formula	-
**Kreissl 2017**	Austria	VLBW	Donor Milk	Preterm formula	-
**Jacobi Pollistok 2016**	USA	VLBW	Human Milk (different percentiles)	Preterm Formula	-
**Paker 2012**	USA	VLBW	>50% Human Milk	Preterm formula	-
**Vohr 2007**	USA	VLBW	Breast Milk	Preterm formula	-
**Huston 2018**	USA	ELBW	Human milk + fortifier	Preterm formula	-
			Human milk	Preterm formula	-
**Ginovart 2016**	Spain	VLBW	Human milk (mother and donor)	Preterm formula	-
**Tanaka 2009**	Japan	Premture	Breast Milk	Preterm Formula	-
**Furman 2003**	USA	VLBW	Breast Milk	Preterm formula	-
**Madore 2017**	USA	VLBW *etnia	Mothers own milk	Donor Breast milk	Preterm formula
**Herrman 2014**	USA	Preterm	Breast milk	Preterm formula	-
**Sisk 2007**	USA	VLBW	< 50% Human Milk	>50% Human Milk	-
**Sisk 2016**	USA	VLBW	< 50% Human Milk	>50% Human Milk	-
**Manea 2018**	Romani	VLBW	Human milk	Preterm formula	
**Chowing 2016**	USA	VLBW	>90% human milk	Preterm Formula	-
**Maayan-Metzger 2012**	Israel	Preterm	>80% human milk	Preterm Formula	
**Assad 2016**	USA	VLBW	Donor Milk	Preterm formula	-
**Giuliani 2012**	Italy	VLBW	Human milk (mother and donor)	Preterm formula	

Table S6. Characteristics of included studies.

Author, year	Human milk and/or breast feeding						Mixed feeding						Preterm formula					
	n	%male	Birth weight	Gestational age	NEC incidence		n	%male	Birth weight	Gestational age	NEC incidence		n	%male	Birth weight	Gestational age	NEC incidence	
					n	%					n	%					n	%
									Observational									
**Verd 2015**	148	58	800	26.4	23	15.4	-	-	-	-	-	-	53	50	830	27.4	11	20.7
**Giuliani 2012**	46	39.1	968	28.2	0	0	-	-	-	-	-	-	46	43.5	984	28.2	1	2.1
**Tanaka 2009**	10	50	1016	28.7	0	0	-	-	-	-	-	-	8	50	1188	30.7	0	0
**Ginovart 2016**	114	48	1078	29.14	11	9.6	-	-	-	-	-	-	72	46	1108	29.5	6	8.3
**Huston 2018**	94		1025	28.4	5	5.3	-	-	-	-	-	-	54		904	26.6	9	16.6
**Berkhout 2018**	631	52	920	26.9	37	5.8	127	52	920	26.9	11	-	103	52	920	26.9	11	10.7
**Bishop 2010**	152	56	1059	28.6	10	6.6	-	-	-	-	-	-	179	48	1056	28.5	12	6.7
**Chowing 2016**	71	-	1016	-	0	0	-	-	-	-	-	-	76	-	1015	-	8	10.5
**Corpeleijn 2012**	300	-	-	-	-	-	300	-	-	-	-	-	49	-	-	-	N.R. (*)-	-
**Spiegler 2016**	223	53	1100	29.0	2	0.9	971	54	1050	-	26	-	239	53	1080	28.7	14	5.8
**Manea 2016**	18	-	-	-	1	5.6	-	-	-	-	-	-	16	-	-	-	2	12.5
**Zamrik 2018**	217	55	892	27	14	6.4	46	55	892	27	7	15.2	171	-	-	-	10	5.8
**Assad 2016**	87	60	-	-	-	-	-	-	-	-	-	-	30	30	-	-		-
**Colacci 2017**	39	-	783	26.0	4	10.2	-	-	-	-	-	-	46	-	770	26.0	5	10.8
**Hair 2016**	819	50.2	844	26.5	56	6.8	-	-	-	-	-	-	768	49.5	823	26.4	128	16.7
**Herrman 2014**	162	55	1361	29.6	7	4.3	-	-	-	-	-	-	443	51	1334	29.7	17	3.1

**Kreissll 2017**	133	-	-	-	12	9.0	-	-	-	-	-		150	-	-	-	8	5.3
**Maayan-Metzger 2012**	188	63.3	1304	30.5	0	0							172	50	1425	31	5	2.9
									**RCT**									
**Cristofalo 2013**	29	41	996	27.7	1	3.4	-	-	-	-	-	-	24	46	983	27.5	5	20.8
**O'Connor 2016**	181		968		7	3.8	-	-	-	-	-	-	182		973		12	6.6
**Sullivan 2010**	141	40	906	26.5	8	5.7	-	-	-	-	-	-	69	52	922	27.3	11	15.9
**Schanler 2005**	151	53	971	27.2	9	6.0	-	-	-	-	-	-	92	46	957	27.2	10	10.9
**Manzoni 2017**	314	53	1125	29.4	20	6.4	-	-	-	-	-	-	184	54	1110	29.2	7	3.8
**Corpeleijn 2016**	183	50.3	1065	28.3	17	9.3	-	-	-	-	-	-	190	54.7	1077	28.6	17	8.9

* statistically significant difference between human milk and preterm formula.

Legend

Selection

1) Is the case definition adequate?
   a) yes, with independent validation ⚜
   b) yes, eg record linkage or based on self reports
   c) no description

2) Representativeness of the cases
   a) consecutive or obviously representative series of cases ⚜
   b) potential for selection biases or not stated

3) Selection of Controls
   a) community controls ⚜
   b) hospital controls
   c) no description

4) <u>Definition of Controls</u>

a) no history of disease (endpoint) ⚜

b) no description of source

Comparability

1) <u>Comparability of cases and controls on the basis of the design or analysis</u>

a) study controls for (Select the most important factor.) ⚜

b) study controls for any additional factor ⚜

Exposure

1) Assessment of Outcome

For some outcomes (e.g. fractured hip), reference to the medical record is sufficient to satisfy the requirement for confirmation of the fracture. This would not be adequate for vertebral fracture outcomes where reference to x-rays would be required.

a) Independent or blind assessment stated in the paper, or confirmation of the outcome by reference to secure records (x-rays, medical records, etc.) ⚜ ☆

b) Record linkage (e.g. identified through ICD codes on database records)☆

c) Self-report (i.e. no reference to original medical records or x-rays to confirm the outcome)

d) No description.

2) Was Follow-Up Long Enough for Outcomes to Occur

a) An acceptable length of time should be decided before quality assessment begins (e.g. 5 yrs. for exposure to breast implants) ⚜

3) Adequacy of Follow Up of Cohorts

a) This item assesses the follow-up of the exposed and non-exposed cohorts to ensure that losses are not related to either the exposure or the outcome.⚜⚜

Allocation of stars as per rating sheet

	ES	95% CI	W	Sig.	N
Colaizy 2012	0.63	0.03 , 12.47	2.85%	0.759	150
Furman 2003	0.17	0.01 , 2.98	3.05%	0.222	119
Jacobi-Pollistok 2016	0.08	0.01 , 0.62	5.93%	0.016	566
O'Connor 2003	0.41	0.05 , 3.54	5.35%	0.416	301
Parker 2012	1.00	0.23 , 4.31	11.13%	1.000	80
Sisk 2009	0.27	0.07 , 0.98	13.98%	0.047	202
Vohr 2006	0.67	0.41 , 1.10	57.71%	0.115	1035
Human Milk (High dose vs Low dose)	0.51	0.31 , 0.85	100.00%	0.010	2453
Berkhout 2018	0.58	0.40 , 0.83	31.41%	0.003	640
Spiegler 2016	0.82	0.63 , 1.06	49.57%	0.135	971
Zamrik 2018	0.87	0.53 , 1.44	19.02%	0.585	346
Human Milk vs Mixed Feeding	0.74	0.59 , 0.94	100.00%	0.014	1957
Assad 2016	0.27	0.13 , 0.59	7.70%	0.001	117
Berkhout 2018	0.31	0.15 , 0.61	8.42%	0.001	631
Bishop 2010	0.99	0.67 , 1.47	11.29%	0.960	331
Chowing 2016	0.42	0.23 , 0.77	9.23%	0.005	147
Colacci 2012	1.07	0.27 , 4.28	3.92%	0.927	85
Ginovart 2016	1.17	0.41 , 3.33	5.69%	0.762	186
Giuliani 2012	0.23	0.03 , 2.17	1.85%	0.201	92
Hair 2016	0.37	0.26 , 0.51	11.85%	0.000	1587
Herrmann 2015	0.18	0.07 , 0.43	6.76%	0.000	1442
Huston 2018	0.28	0.09 , 0.89	5.05%	0.031	148
Kreissl 2017	0.64	0.24 , 1.67	6.21%	0.362	283
Madore 2017	0.65	0.10 , 4.16	2.52%	0.650	81
Manea 2016	5.00	0.22 , 112.34	1.02%	0.311	34
Mayaan-Metzger 2012	0.09	0.00 , 1.89	1.09%	0.123	86
Spiegler 2016	0.15	0.03 , 0.65	3.53%	0.011	462
Tanaka 2009	0.81	0.01 , 45.22	0.63%	0.918	18
Verd 2015	0.70	0.32 , 1.56	7.47%	0.386	201
Zamkir 2018	0.33	0.12 , 0.94	5.76%	0.037	474
Human Milk vs Preterm Formula (Observational Studies)	0.45	0.32 , 0.62	100.00%	0.000	6405
Corpeleijin 2016	0.96	0.47 , 1.94	17.46%	0.909	373
Cristofalo 2013	0.46	0.17 , 1.24	11.40%	0.126	53
Manzoni 2013	0.81	0.58 , 1.13	30.16%	0.220	498
O'Connor 2016	0.33	0.13 , 0.79	13.29%	0.013	363
Schanler 2005	0.25	0.09 , 0.69	10.80%	0.008	170
Sullivan 2010	0.89	0.43 , 1.84	16.89%	0.753	169
Human Milk vs Preterm Formula (Trial)	0.62	0.42 , 0.93	100.00%	0.020	1626
Berkhout 2018	1.27	0.80 , 2.00	16.94%	0.307	245
Corpeleijn 2012	1.73	1.03 , 2.92	13.10%	0.039	0
Spiegler 2016	1.37	1.06 , 1.77	54.11%	0.016	1210
Zamkir 2018	1.22	0.76 , 1.96	15.85%	0.411	217
Mixed Feeding vs Preterm Formula	1.37	1.13 , 1.65	100.00%	0.001	1672

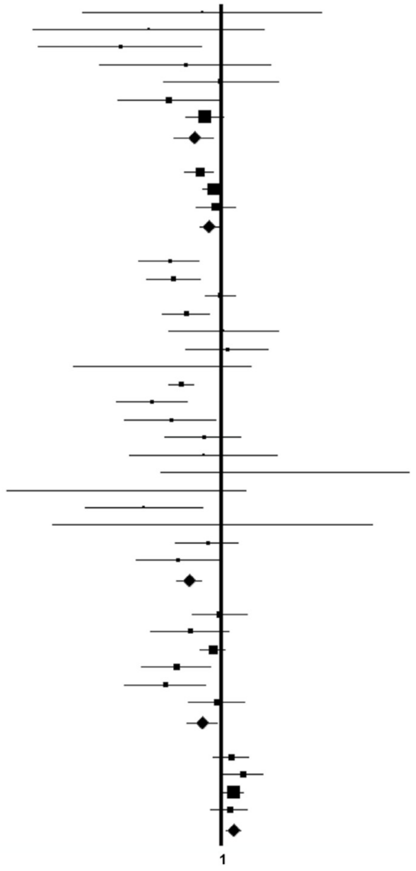

Figure S1. Meta-analysis: results from RCT and observational studies. Forest plot for selected outcomes.

Table S7. Human Milk Banks in the world

**Nation**	**HMB**	**Nation**	**HMB**
Afghanistan	0	Cyprus	0
Albania	0	Czechia	4
Algeria	0	Democratic People's Republic of Korea	0
Angola	0	Democratic Republic of the Congo	0
Antigua and Barbuda	0	Denmark	2
Argentina	5	Djibouti	0
Armenia	0	Dominican Republic	1
Australia	5	Ecuador	0
Austria	2	Egypt	0
Azerbaijan	0	El Salvador	0
Bahamas	0	Equatorial Guinea	0
Bahrain	0	Eritrea	0
Bangladesh	0	Estonia	1
Barbados	0	Ethiopia	0
Belarus	0	Fiji	0
Belgium	4	Finland	17
Belize	0	France	36
Benin	0	Gabon	0
Bhutan	0	Gambia	0
Bolivia (Plurinational State of)	1	Georgia	0
Bosnia and Herzegovina	0	Germany	20
Botswana	0	Ghana	0
Brazil	214	Greece	2
Brunei Darussalam	0	Grenada	0
Bulgaria	1	Guatemala	1
Burkina Faso	0	Guinea	0
Burundi	0	Guinea-Bissau	0
Cabo Verde	1	Guyana	0
Cambodia	0	Haiti	0
Cameroon	0	Honduras	0
Canada	4	Hungary	8
Central African Republic	0	Iceland	2
Chad	0	India	22
Chile	1	Indonesia	0
China	14	Iran (Islamic Republic of)	1
Colombia*	5	Iraq	0
Comoros	0	Ireland	1
Congo	0	Israel	1

Costa Rica	2	Italy	37
Côte d'Ivoire	0	Jamaica	1
Japan	1	Papua New Guinea	0
Jordan	0	Paraguay	1
Kazakhstan	0	Peru	1
Kenya	1	Philippines	0
Kingdom of Eswatini	0	Poland	11
Kiribati	0	Portugal	1
Kuwait	1	Qatar	0
Kyrgyzstan	0	Republic of Korea	1
Lao People's Democratic Republic	0	Republic of Moldova	0
Latvia	0	Romania	0
Lebanon	0	Russian Federation	2
Lesotho	0	Rwanda	0
Liberia	0	Saint Lucia	0
Lithuania	2	Saint Vincent and the Grenadines	0
Luxembourg	0	Samoa	0
Madagascar	0	Sao Tome and Principe	0
Malawi	0	Saudi Arabia	0
Malaysia	0	Senegal	0
Maldives	0	Serbia	3
Mali	0	Seychelles	0
Malta	1	Sierra Leone	0
Mauritania	0	Singapore	1
Mauritius	0	Slovakia	6
Mexico	8	Slovenia	1
Micronesia (Federated States of)	0	Solomon Islands	0
Mongolia	0	Somalia	0
Montenegro	0	South Africa	44
Morocco	0	South Sudan	0
Mozambique	1	Spain	15
Myanmar	0	Sri Lanka	0
Namibia	1	State of Libya	0
Nepal	1	Sudan	0
Netherlands	1	Suriname	0
New Zealand	1	Sweden	28
Nicaragua	3	Switzerland	7
Niger	0	Syrian Arab Republic	0
Nigeria	1	Tajikistan	0
Norway	12	Thailand	0
Pakistan	1	The former Yugoslav republic of Macedonia	0

Panama	1	Timor-Leste	0
Trinidad and Tobago	0	United States of America***	24
Tunisia	0	Uruguay	0
Turkey	0	Uzbekistan	0
Turkmenistan	0	Vanuatu	0
Uganda	0	Venezuela (Bolivarian Republic of)	10
Ukraine	1	Viet Nam	1
United Arab Emirates	0	Yemen	0
United Kingdom of Great Britain and Northern Ireland**	16	Zambia	0
United Republic of Tanzania	0	Zimbabwe	0
